# Association of Two Opposing Responses Results in the Emergence of a Novel Conditioned Response

**DOI:** 10.3389/fnbeh.2022.852266

**Published:** 2022-04-29

**Authors:** Micaela R. Pribic, Aristide H. Black, Asia D. Beale, Jessica A. Gauvin, Lisa N. Chiang, Jacqueline K. Rose

**Affiliations:** ^1^Biology Department, Western Washington University, Bellingham, WA, United States; ^2^Department of Psychology, Western Washington University, Bellingham, WA, United States

**Keywords:** opposing responses, *C. elegans*, delayed conditioning, photosensory, mechanosensory, classical conditioning

## Abstract

Recent studies examining association of opposing responses, contrasting emotional valences, or counter motivational states have begun to elucidate how learning and memory processes can translate to clinical therapies for trauma or addiction. In the current study, association of opposing responses is tested in *C. elegans*. Due to its relatively simple and well-described nervous system, it was hypothesized that association of two oppositional stimuli presented in a delayed conditioning protocol would strengthen the behavioral response to the first stimulus (alpha conditioning). To test this, *C. elegans* were exposed to a tone vibration stimulus (to activate a mechanosensory-driven locomotor reversal response) paired with a blue light (to activate a forward locomotor response) at a 2-s delay. After five pairings, behavior was measured following a tone-alone stimulus. Worms that received stimulus pairing did not show an enhanced response to the first presented stimulus (tone vibration) but rather showed a marked increase in time spent in pause (cessation of movement), a new behavioral response (beta conditioning). This increase in pause behavior was accompanied by changes in measures of both backward and forward locomotion. Understanding the dynamics of conditioned behavior resulting from pairing of oppositional responses could provide further insight into how learning processes occur and may be applied.

## Introduction

Modifying behavior by conditioning a new response has numerous clinical applications. A “strong” memory like fear may be considered more resistant to disruption or alteration due to learning (Roozendaal and McGaugh, 2011). Recent work has attempted to counter-condition strong fear responses (anxiety disorder, post-traumatic stress disorder; PTSD) or drug cravings for the treatment of addiction. For instance, Hurd et al. (2019) reported a reduction in cue-induced craving and behavioral indicators of anxiety (increased heart rate, temperature and salivary cortisol levels) following repeated presentation of cannabidiol to counter feelings of stress brought on by heroin drug cues in opioid addicts (Fatseas et al., 2011; Preston et al., 2018). Using a similar rationale, better treatment outcomes for PTSD patients are reported when 3,4-methylenedioxymethamphetamine (MDMA) is delivered during psychotherapy sessions to counter the anxiety and/or fear-responses activated during recall of traumatic memories (Mithoefer et al., 2019; Mitchell et al., 2021). At the simplest level, the approach of driving an opposing behavioral response (even if pharmacologically induced) to alter future behavior can be seen as a function of signal integration; thus, it is of interest to investigate how signal integration occurs in a nervous system.

In rodent models, conditioning of opposing responses has been tested by pairing two unconditioned stimuli (US-US conditioning); for instance, rats salivating in response to a footshock following food-shock pairing (Gormezano and Tait, 1976). However, there are few reports of this form of conditioning. More often, investigators counter-condition a previously conditioned response. For example, rabbits conditioned to perform an appetitive jaw-movement following tone-water conditioning were later trained with water-pre-orbital shock pairings and showed both jaw-movement and nictitating membrane responses to tone suggesting parallel opposing response pathways were activated to the conditioned tone stimulus (Tait et al., 1986). More recently it has been purported that individual neurons can be involved in driving opposing responses as mice conditioned to escape following photostimulation of neurons expressing channelrhodopsin (ChR2) in piriform cortex, will show a subsequent appetitive licking response when ChR2 activation of the same neurons is later paired with water presentation (Choi et al., 2011). A circuit-specific investigation of conditioning of opposing responses could elucidate the learning processes involved.

Part of the challenge to uncovering plasticity processes involved with conditioning opposing response behaviors is due to the complexity of the nervous systems employed. *Caenorhabditis elegans* (*C. elegans*) is a microscopic nematode with a neural connectome made up of 302 neurons (White et al., 1986). These neurons and their connections have been identified (Cook et al., 2019). A popular example of conditioning opposing outcomes in *C. elegans* is to generate an avoidance response to the attractant sodium chloride (NaCl) by pairing NaCl with the absence of food (Saeki et al., 2001). The same avoidance response can be conditioned to other water-soluble attractants as well [cyclic adenosine monophosphate (cAMP), biotin, lysine; Saeki et al., 2001]. Other examples of conditioning avoidance behavior to attractant signals in *C. elegans* include pairing cultivation temperature with starvation, or 1-propanolol with an aversive acidic pH environment produced by hydrochloric acid (Mohri et al., 2005; Amano and Maruyama, 2011). Some have also shown that an aversive stimulus (1-nonanol or ultraviolet light) can be conditioned to drive an appetitive response when paired with an attractant (potassium chloride or food; Nishijima and Maruyama, 2017; Ozawa et al., 2022). Thus, *C. elegans* are capable of updating response circuits when stimuli are presented with opposing conditions.

**FIGURE 1:**
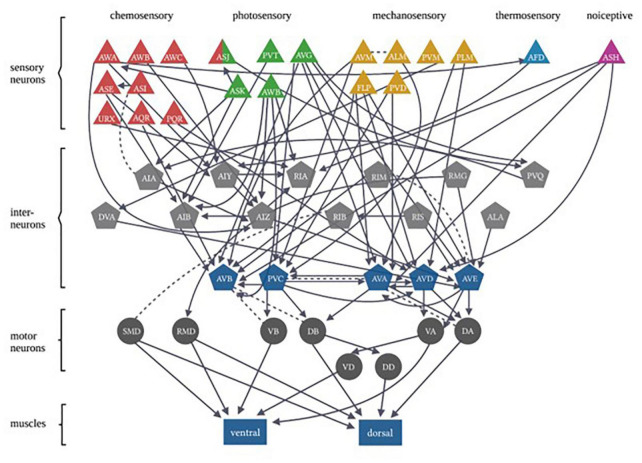
*Caenorhabditis elegans* photo- and mechanosensory neural circuits are embedded within a broader set of multiple sensory-response circuits. Sensory neurons are represented by triangles, with colors differentiating between different sensory inputs. Gray pentagons represent interneurons and blue pentagons represent command interneurons. Motor neurons are indicated by circles that extend connections to body-wall muscles represented by rectangles (Figure adapted from Fang-Yen et al. (2015), with permission from the Royal Society, United Kingdom).

In each of these examples of *C. elegans* opposing state conditioning, the conditioned response activates the same forward locomotion neural circuitry in order to produce differential migration patterns (i.e., forward movement toward or away from the conditioned stimulus). The aim of the current study was to investigate response conditioning by associating two stimuli that drive opposing locomotor responses: generalized mechanosensory stimulus (vibration produced by a tone; Chen and Chalfie, 2014) and a blue light stimulus. Worms typically respond to a non-localized mechanosensory stimulus (e.g., tap to the side of the petri dish) with backward locomotion (Wicks and Rankin, 1995). Conversely, worms respond with forward locomotion in response to whole-body blue light illumination (Edwards et al., 2008). The neural circuitry for responses driven by each of these forms of stimuli has been largely identified (see Figure 1) and includes the potential for signal integration at several interneurons (see Table 1). Using a delayed conditioning protocol, it was expected that paired presentation of a tone (vibration) stimulus with a blue light would result in a strengthening of the mechanosensory reversal response as the onset of the blue light presentation was delayed. However, responses to both stimuli appeared to be modulated following conditioning.

**TABLE 1:** Neurons involved in photosensory processing.

Photosensory neurons	PVT	AVG	ASJ	ASK	AWB
Interneurons	**RIG** **RIH** **AVK** DVC **AIB**	**PVC** **PVP** PVQ PVT **AVA** **AVB** **AVD** **AVE** **AVJ**	AIM PVQ	**AIA** **AIB** **RIF** AIM	**AIB** **AIZ** **AVB** **RIA** RIR
Interneurons/motor neurons	**SMB** AVL RMF	PVN AVF AVL	AVF		**SMB**
Motor neurons	RME	VA DA	**HSN**		
Sensory neurons		PHA	**ASK**	**CEP** AWA ASJ	**ADF** **ASG** **ASH**

*Bold indicates neurons also have a reported role in detecting and responding to mechanosensory stimuli (compiled from Chalfie et al., 1985; Von Stetina et al., 2006; Ward et al., 2008; Haspel et al., 2010; Bhatla and Horvitz, 2015).*

## Methods

### Strains and Strain Maintenance

N2 (Wild Type, acquired originally from the *Caenorhabditis* Genetics Center, U Minnesota) colonies were maintained on NGM agar plates at 20°C and fed the OP50 *E. coli* strain (Stiernagle, 2006). Worm colonies were age-synchronized, cultivated at 20°C and tested 4 days later (day L4 + 1). This adult stage was chosen for testing to ensure complete maturation of mechanosensory neurons, as the touch-sensitive AVM neuron matures in a late larval stage (Chalfie et al., 1985). Age synchronization entailed picking 10–12 adult worms and submersing them in a ∼5 ul drop of 2:1 bleach to 1 M NaOH solution on a seeded plate and testing surviving progeny (see Stiernagle, 2006; Meneely et al., 2019).

### Behavioral Assay and Apparatus

**FIGURE 2:**
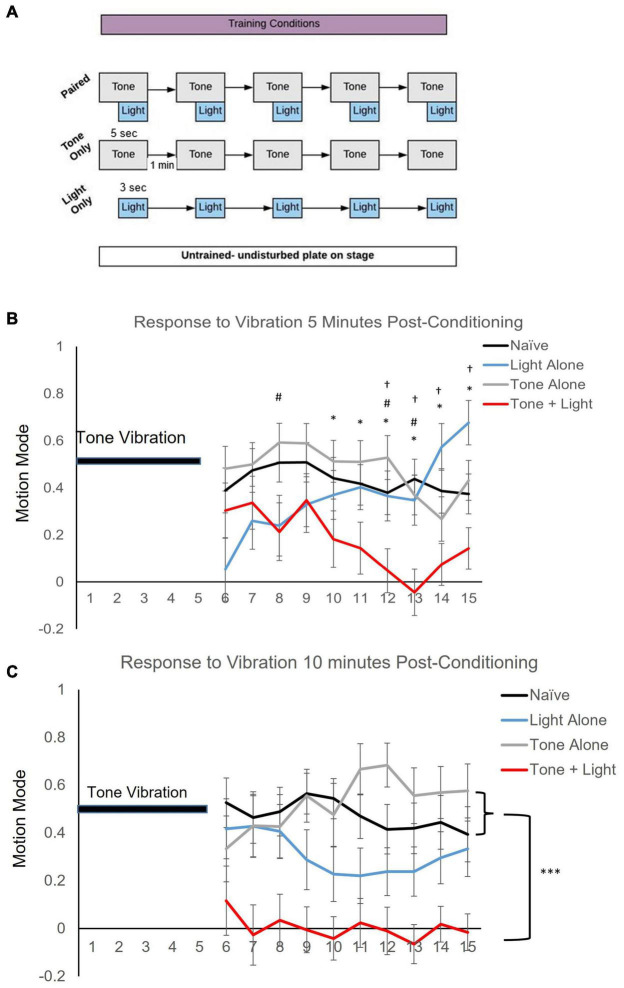
Training condition affects locomotor direction of worms following a single tone stimulus. **(A)** Training protocol showing Naïve (untrained), Tone Alone, Light Alone and Paired conditioning. Stimulus pairing consisted of five presentations of a 5-s tone vibration stimulus delivered with a 3-s blue light stimulus at a 2-s delayed onset. Testing consisted of a single 5-s tone stimulus. **(B)** Mean motion mode (± SEM) (indicates average vector of movement direction whereby + values signify forward and − values signify backward locomotion direction) captured for the 10-s period following the 5-s test tone at 5 min after conditioning. *Post-hoc* analyses indicate: *p < 0.05 between Naïve (*n* = 46) and Paired (*n* = 29); ^#^*p* < 0.05 between Tone Alone and Paired (*n* = 25); ^†^*p* < 0.05 between Light Alone (*n* = 30) and Paired training conditions. **(C)** Mean motion mode (± SEM) captured for a 10-s period following a 5-s test tone at 10 min after conditioning. *Post-hoc* analyses indicate: ^***^p < 0.001 of main effects.

Behavioral training and testing was conducted on seeded NGM plates with ∼20 worms per plate. During testing, on average ∼10 worms were video captured per plate. Training and testing were conducted in the dark with blue light filters on all computer monitors and the microscope stage, to control for blue light from other light sources. Worms were acclimated to the training/testing conditions for 2 min prior to any stimulus delivery. Worms in the Naïve group were positioned in the training/testing apparatus for an equal period of time as the stimulus conditions but Naïve received no blue light or tone stimuli. The conditioning assay consisted of five pairings of a 5-s 300 Hz tone vibration, produced by placing a speaker (XM12001 X-Vibe 3.0 Vibration Speaker by XDream) next to and in contact with the worm-containing agar plate on the microscope stage (Olympus SZ7), with 2-s delayed onset of a 3-s 470 nm blue light stimulus at 1,000 mA (Mightex LED) placed above the plate. Training stimuli were presented at a 1-min interstimulus interval (ISI). For the Tone Alone condition, worms were exposed to five presentations of the 5-s tone stimulus at a 1-min (ISI) and for the Light Alone condition worms were exposed to the 3-s blue-light stimulus at a 1-min ISI (see Figure 2A). Testing consisted of a 5-s tone vibration stimulus alone delivered at 5 and 10 min after the last pairing or following a comparable time period on the microscope for the Naïve group.

**FIGURE 3:**
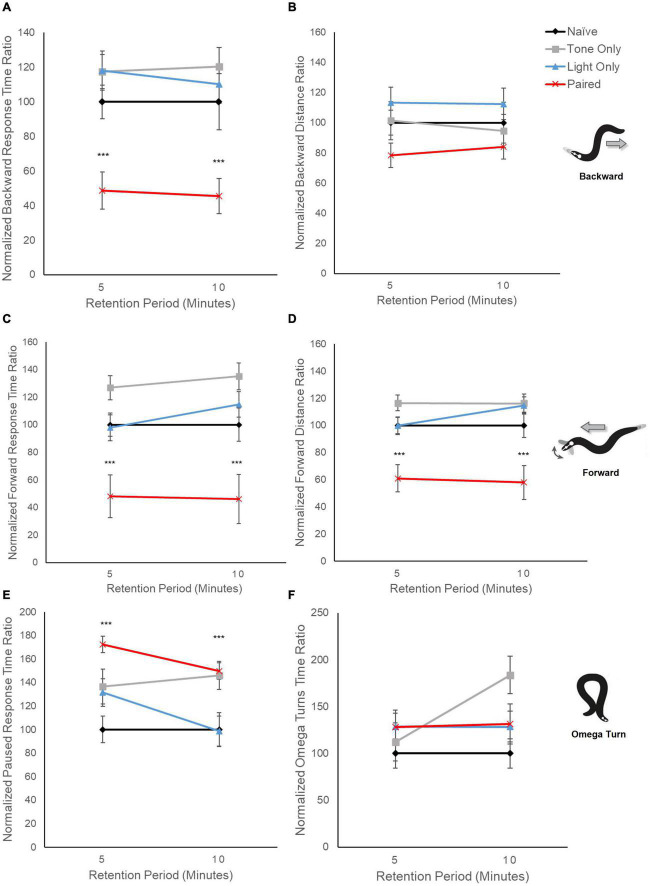
Locomotion metrics indicate differences in locomotor response circuits following conditioning. Graphs display averaged response metrics for a period of 60 s following a 5-s tone stimulus delivered at 5 min and 10 min after conditioning (retention period). Average response metrics of all training groups are expressed relative to the average of Naïve worm responses. Mean (± SEM) for: **(A)** backward response time ratio, **(B)** backward distance ratio, **(C)** forward time ratio, **(D)** forward distance ratio, **(E)** paused time ratio, and **(F)** omega turn time ratio. ***p < 0.001 between Naïve and Paired groups. Worm movement illustrations adapted with permission from Broekmans et al. (2016).

Video of the locomotor response to tone vibration was captured *via* CCD Camera (AmScope MUB2003). Videos of behavior were captured for the duration of stimulus presentation plus 10 or 60 s. Behavior was only analyzed for the periods after stimulus presentation because of minor video distortion due to vibration during stimulus presentation, so stimulus offset was used to consistently mark the beginning of analyses. Each trial had a minimum of three replicates and each group had trials that were tested over a minimum of three different days with trained and untrained matched controls for every test day to randomize any effects due to environmental fluctuations. For the motion mode analysis (Figure 2) data was captured from a total of 130 worms with 5 worms not responding for the recording period. For locomotor metrics (Figure 3), data was derived from 1,327 different worm trajectories. Data recorded at 5 and 10 min post-training were captured from the same plates of worms for both time points. No worms were excluded from analysis.

### Behavior Analysis

All behavior videos were analyzed using TierPsy Multi-Worm tracker (V1.4.0; Javer et al., 2018a,b). This program is a free download, found here: https://github.com/ver228/tierpsy-tracker. Descriptions of output files for TierPsy are found here: https://github.com/ver228/tierpsy-tracker/blob/master/docs/OUTPUTS.md.

#### Tierpsy Criteria for Locomotor Responses

Forward: The animal is moving in the “head direction” for at least 0.5 s and at least 5% of its length.

Backward: The animal is moving in the “tail direction” for at least 0.5 s and at least 5% of its length.

Pause: The animal is moving neither in the head nor tail direction for at least 0.5 s.

Omega Turns: The animal moves forward; the head side then turns back at a sharp angle to become even with the tail and swims off in the direction at which the animal was coming from in a forward motion.

#### Behavior Metrics

Descriptions of the calculated locomotor measures provided in Tierpsy software:

Time Ratio: (no units) ratio between the time spent at the event over the total trajectory time. This is calculated for forward, reversal, pause, and omega turn behaviors.

Distance Ratio: (no units) ratio between the total distance traveled during an event and the total distance traveled during the whole trajectory. This is calculated for only forward and reversal behaviors.

Motion Mode: vector indicating if the worm is moving forward (1), backward (−1), or is paused (0). This measure is unique for every animal, as it assigns a number for the above movements for every frame that animal is in.

### Statistical Analysis

Analysis of behavioral data was performed using Mixed repeated measures ANOVAs for motion mode with least significant difference for *post-hoc* analyses (*p* < 0.05 significance). Locomotor metrices were analyzed with two-way ANOVAs, with Dunnett’s *post-hoc* comparisons to determine the effect of training, by comparing the results of vibration-light training, light-only and tone-only to the appropriate untrained group, of the same retention period (*p* < 0.05 significance). Analyses were completed in R, using package “car” for analysis and “ggplot2” for data visualizations or IBM SPSS Statistics (Ver. 26, Microsoft). For response metrics, visualization of behavioral data for trained, light-only, or tone-only animals is normalized to data from untrained animals (% Naïve) and presented as normalized mean. Lucidchart, Adobe Illustrator and Excel were used to create diagrams and schematics.

## Results

Repeated pairings of a mechanosensory tone stimulus with a blue light stimulus result in a difference in response to the tone stimulus alone. Motion mode (vector indicator where + values indicate forward motion and−values indicate backward motion) was captured for a 10-s period after a vibration test stimulus presentation. Results show a statistically significant interaction between training and time on locomotion direction at 5 min post-training (*F*_3.817, 11.452_ = 3.478, *p* < 0.001 with Greenhouse-Geisser correction; Figure 2B). Despite the variability of behavior across trials from animals in all training conditions in the initial seconds following delivery of the tone stimulus, only the Paired group shows locomotion direction at ∼0 (meaning worms were not moving forward or backward, but were likely paused) for the latter time-period measured (5–10 s after tone stimulus delivery). At 10 min after conditioning, there was no interaction of training across time (*F*_10.101,420.880_ = 1.013, *p* > 0.05) as there was no statistical difference in response direction over time (*p* > 0.05); however, there was a significant effect of training condition (*p* < 0.001). Multiple comparisons across training conditions (corrected for multiple tests) indicate significant differences between the Paired group and both the Naïve (*p* < 0.001) and the Tone Alone (*p* < 0.001) groups. Multiple comparisons did not reveal a significant difference between the Light Alone group and any of the other training conditions (*p* > 0.05) though there was a trend toward significance between the Paired and Light Alone group (*p* = 0.076; Figure 2C). These data indicate that pairing of a tone vibration with a blue light stimulus to drive opposing locomotor responses affects subsequent responding to the tone stimulus.

Examination of distinct locomotor response elements provides some indication as to how behavior has changed following conditioning. When the relative time spent performing backward locomotion is captured and averaged over a longer period following the mechanosensory tone stimulus presentation (60 s), trained worms show a notable decrease compared to control groups (*F*_3,633_ = 26.30, *p* < 0.001; Figure 3A). This overall difference in response behavior cannot be accounted for by pseudoconditioning to either the tone stimulus or the light stimulus alone as worms repeatedly exposed to a single tone stimulus or light stimulus did not show the same decline in backward relative response time (*p* > 0.10). Interestingly, the decrease in backward locomotion response time did not correspond to a significant decrease in relative backward distance traveled (*F*_3,483_ = 2.230, *p* = 0.08; Figure 3B). For forward locomotion, the relative amount of time trained worms spent moving in a forward direction was also significantly decreased across retention periods compared to the control groups (*F*_3,633_ = 45.11, *p* < 0.001; Figure 3C); however, a significant decrease in the relative distance traveled while moving forward was also noted for the trained group (*F*_3,633_ = 43.72, *p* < 0.001; Figure 3D). Together, these data suggest that responding in the mechanosensory-driven reversal circuit and the light-driven forward circuit appear altered following conditioning.

As earlier data indicated consistent average motion mode direction around zero in trained worms (see Figures 2B,C), we also examined relative time spent performing pause behavior (neither forward nor backward locomotion) and found a significant increase in paused behavior response time ratio for trained worms compared to controls (*F*_3,557_ = 6.46, *p* < 0.001; Figure 3E). This is interesting as it could suggest that neither locomotor circuit is activated following conditioning, or conversely that both are now activated, and the effects cancel out. The tone-only experimental group also showed a significant increase in relative pause time but only at the 10-min retention interval (*p* < 0.001).

To probe if the increase in paused behavior and decrease in both backward and forward locomotion could reflect an overall change in locomotor responding a separate avoidance behavior metric was analyzed across groups. Omega turns are avoidance responses whereby worms reverse into a circular body position in order to change direction. Interestingly, there appeared to be no significant difference in the time spent performing Omega turns across groups (*F*_3,347_= 1.99, *p* = 0.11) suggesting the effects on locomotor behavior are not due to a generalized locomotor deficit.

## Discussion

Associative conditioning of opposing response circuits results in a change in locomotor behavior following delivery of a single tone test stimulus (Figures 2B,C). As well, the shift to a pause response to the tone vibration appears to become more consistent at 10 min after conditioning compared to 5 min. As the same worm plates are tested at both time points, it is possible that learning increases by 10 min post-conditioning if the tone-CS presentation at 5 min serves as reminder (Spear, 1973). Other work in *C. elegans* has shown enhanced learning following subsequent presentation of learning stimuli (Rose et al., 2002; Amano and Maruyama, 2011). It is also possible that some gradual plasticity process or memory consolidation mechanism is activated following the repeated pairings. Examples of this include mitogen-activated protein kinase (MAPK)-dependent sensitization of the siphon withdrawal reflex in *Aplysia* following repeated shock delivery (Sutton et al., 2002: Sharma et al., 2003), increased calcium levels in associated neurons after odor-shock pairing in *Drosophila* (Wang et al., 2008), and time-dependent enhancement of contextual fear memory when multiple shocks are given during conditioning (Poulos et al., 2016).

It was anticipated that initial onset of the tone vibration stimulus would prime any response modulation to be entrained only to the tone as response modification with US-US delayed conditioning should result in the first stimulus being influenced by the second stimulus in the pairing (Schreurs and Alkon, 1990). Preliminary data from trials where the blue light was presented first with delayed tone onset suggest that tone presentation could modulate response to blue light in a similar way (see Supplementary Figures 1A–C) though technical challenges limit interpretation of these data (see Supplementary Material). As there are a number of neurons included in both the mechanosensory and the photosensory neural circuits (see Table 1), it is possible that repeated pairing stimulates some plasticity mechanism that alters the response probabilities for both stimuli.

When backward and forward locomotion were analyzed separately, trained worms showed significant decreases in the relative time spent performing either forward or backward locomotion following a tone vibration test stimulus compared to naïve and to single-stimulus training groups (Figures 3A,C). Interestingly, trained worms also showed a significant increase in relative time spent paused (no locomotion; Figure 3E). In rodents, individual neurons in olfactory piriform cortex can be involved in both appetitive and avoidance responses such that when a neuron activated with an aversive outcome is re-activated in an appetitive environment neither the appetitive nor avoidance behavior occurs but instead a freezing response is initially seen (Choi et al., 2011). Thus, it is possible that the immediate effect of co-activation of opposing response circuits is a similar “freezing” response in the worm. Future research will need to examine further how this cessation in locomotion occurs following conditioning as well as determine the duration this increase in pause behavior persists.

As mentioned, there are both independent and overlapping neurons included in both the mechanosensory and photosensory neural circuitry (Figure 1). The majority of neurons involved in both circuits are interneurons (Table 1), in particular, all of the locomotor command interneurons (AVA, AVD, AVE, AVB, PVC) thus again identifying these neurons as possible sites for neural signal integration leading to a decision of locomotor response behavior (Chalfie et al., 1985; Piggott et al., 2011; Kaplan et al., 2018). In mammals, the ventral pallidum has been reported to contain both positive- and negative-valence specific neurons that differentially influence what behavior is expressed in response to environmental conditions (Stephenson-Jones et al., 2020). In *C. elegans*, behavioral flexibility derived from signal integration (described as a “hub and spoke”’ model) has been reported for multiple sensory systems (Macosko et al., 2009; Rabinowitch et al., 2013; Summers et al., 2015). This form of neural signaling architecture has been previously reported to mediate responses driven by opposing outcomes (threat vs. reward) in worms (Ghosh et al., 2016). Plasticity within a hub interneuron could explain the ability for rapid response modulation following co-activation of the opposing locomotor response circuitry.

There are many questions that remain unanswered with regards to how organisms process and reconcile information from competing and oppositional stimuli. From the current study, future research will need to uncover the extinction parameters for each stimulus, the duration for which this learning persists, and address if additional pairings prolong retention. As well, determining if this form of learning is vulnerable to a stimulus pre-exposure effect that could reduce the efficacy of the association conditioning protocol (Randich and LoLordo, 1979). Finally, it is of great interest to delve in to the neural signaling mechanisms that underlie learning resulting from the co-occurrence of competing inputs. It is likely that similar to other models as well as previous reports of *C. elegans* learning that glutamate signaling and perhaps calcium/calmodulin-dependent kinase II (CaMKII) activation is involved (Amano and Maruyama, 2011; Stein and Murphy, 2014; Ozawa et al., 2022). Given the simplicity of the approach and the relatively rapid conditioning time scale, it may be possible to employ neuron activation tools to capture signaling changes in real-time allowing us to answer many questions about how neurons integrate competing signals to guide behavior. The implications of these results could offer insight into the mechanisms and the efficacy of this conceptual approach in applied settings.

## Data Availability Statement

The raw data supporting the conclusions of this article will be made available by the authors, without undue reservation.

## Author Contributions

MP performed the experiment and analyzed the data except for the testing in response to light stimulus and composed sections of the initial draft. AHB performed the testing in response to the light experiment and analyzed the data. ADB composed multiple sections of the initial draft and compiled Figure 1 and Table 1. JG and LC composed sections of the initial draft. JR conceptualized the study, consulted and supervised on data collection and analysis, and composed and refined all of the manuscript drafts. All authors contributed to the article and approved the submitted version.

## Conflict of Interest

The authors declare that the research was conducted in the absence of any commercial or financial relationships that could be construed as a potential conflict of interest.

## Publisher’s Note

All claims expressed in this article are solely those of the authors and do not necessarily represent those of their affiliated organizations, or those of the publisher, the editors and the reviewers. Any product that may be evaluated in this article, or claim that may be made by its manufacturer, is not guaranteed or endorsed by the publisher.
