# Supplementary figures and images for: Association of Two Opposing Responses Results in the Emergence of a Novel Conditioned Response
Source: Front Behav Neurosci. 2022 Apr 29;16:852266. doi: 10.3389/fnbeh.2022.852266 (PMC9102977; doi:10.3389/fnbeh.2022.852266)

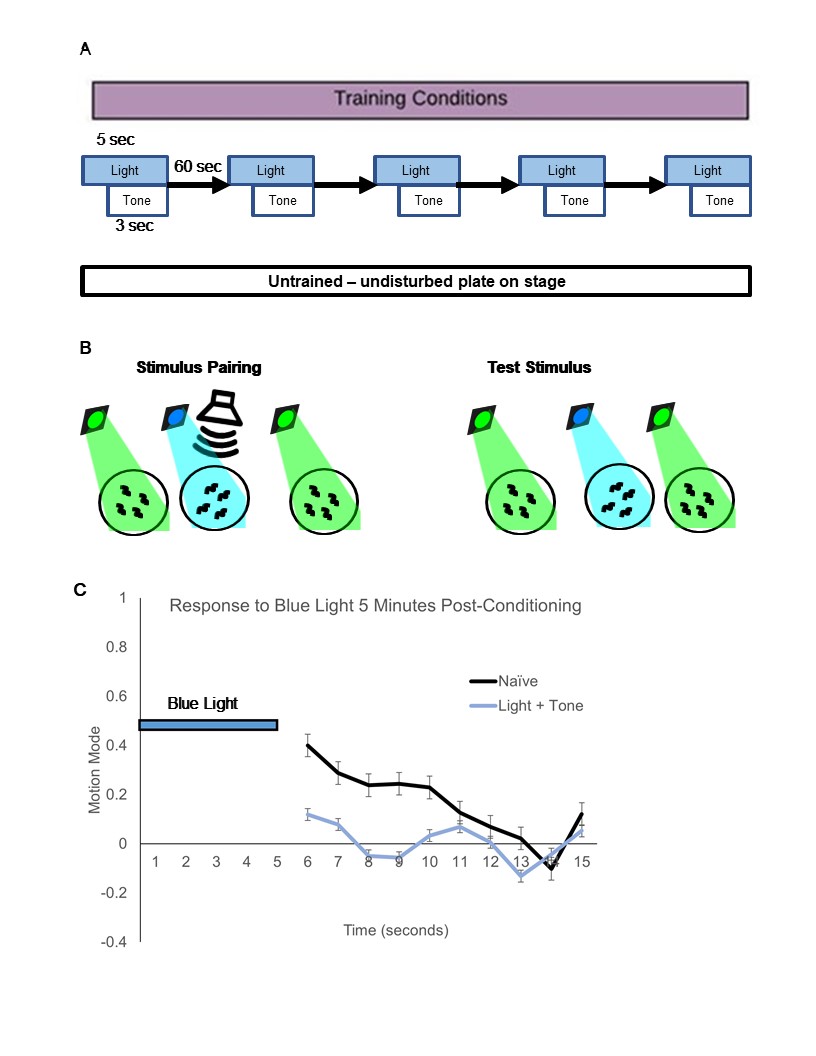

Supplement: Supplementary file 1 [file Image_1.JPEG]
