# Supplementary material for: Association of Two Opposing Responses Results in the Emergence of a Novel Conditioned Response
Source: Front Behav Neurosci. 2022 Apr 29;16:852266. doi: 10.3389/fnbeh.2022.852266 (PMC9102977; doi:10.3389/fnbeh.2022.852266)
Supplement: Supplementary file 2 [file Table_1.DOCX]

Introduction and Method Variation

To test if delayed tone vibration presentation would similarly affect the response to blue light, a trial of reverse pairings was conducted with a delayed conditioning protocol where blue light onset preceded tone vibration onset by two seconds and the two stimuli co-terminated (Supplemental Figure 1A). Capture of behavioral data was impeded as it required at least one second for the camera to respond to the change in light levels before video was acquired. To prevent this delay, green light was used as the default illumination as it had been reported that *C. elegans* are not as responsive to green light wavelengths (Edwards et al., 2008) and pilot studies for the current experiment did not show responses to green light (data not shown). Therefore, when blue light served as the test stimulus, green light (530 nm) illumination (1000mA (Mightex LED) occurred prior to and following the blue light stimulus to avoid interference with video capture due to the camera adjusting to changing light levels (Supplemental Figure 1B).

Results and Discussion

The resultant response data to blue light indicated less forward locomotion when blue light was paired with tone vibration (Supplemental Figure 1C); however, the Naïve worm response to the blue light also showed a decline over seconds that eventually matched the trained worms. Because of the modified testing conditions involving green light, it is difficult to discern why naïve worms were gradually pausing after blue light presentation, particularly since additional reports indicate worms likely detect and may demonstrate light avoidance to longer wavelengths of light, including green light (Ward et al., 2008; Ghosh et al., 2021). For these reasons, response to blue light was not investigated further in this study.

Figure Caption

Supplemental Figure 1. Blue light-tone pairing results in increased paused behavior at 5 minutes post-conditioning. A) Delayed forward conditioning protocol whereby blue-light onset precedes tone onset by 2 sec with stimuli co-terminating for each pairing. B) Lighting conditions for stimulus pairing and for test stimulus delivery highlighting the use of green-light when blue light illumination absent. C) Mean (±SEM) Motion Mode across a 10-second period immediately following blue-light (CS) presentation at 5 minutes post-conditioning.

References:

Edwards, S. L., Charlie, N. K., Milfort, M. C., Brown, B. S., Gravlin, C. N., Knecht, J. E., & Miller, K. G. (2008). A novel molecular solution for ultraviolet light detection in Caenorhabditis elegans. PLoS biology, 6(8), e198.

Ghosh, D. D., Lee, D., Jin, X., Horvitz, H. R., & Nitabach, M. N. (2021). C. elegans discriminates colors to guide foraging. Science, 371(6533), 1059-1063.

Ward, A., Liu, J., Feng, Z., & Xu, X. Z. (2008). Light-sensitive neurons and channels mediate phototaxis in C. elegans. Nature neuroscience, 11(8), 916-922.
